# Mass spectrometry data on volatile compounds of *Polygonum minus* Huds. leaf essential oil

**DOI:** 10.1016/j.dib.2025.111871

**Published:** 2025-07-10

**Authors:** Muhammad Aqil Fitri Rosli, Sharifah Nabihah Syed Jaafar, Kamalrul Azlan Azizan, Salmah Yaakop, Wan Mohd Aizat

**Affiliations:** a Institute of Systems Biology (INBIOSIS), Universiti Kebangsaan Malaysia (UKM), 43600 UKM Bangi, Selangor, Malaysia; b Department of Applied Physics, Faculty of Science and Technology, Universiti Kebangsaan Malaysia (UKM), 43600 UKM Bangi, Selangor, Malaysia; c Centre for Insect Systematics, Faculty of Science and Technology, Universiti Kebangsaan Malaysia (UKM), 43600 UKM Bangi, Selangor, Malaysia

**Keywords:** Kesum, Volatile metabolites, Hydro-distillation, Medicinal plant, GC-MS analysis, Metabolomics

## Abstract

*Polygonum minus* Huds., commonly referred to as kesum, is a traditional medicinal plant in Malaysia known for its unique fragrance attributed to its volatile compounds. The essential oil extracted from *P. minus* displayed various benefits, comprised of antioxidant, antimicrobial, and anticancer properties. Therefore, to identify volatile metabolites associated with such biological activities, we performed untargeted metabolite analysis on the essential oil extracted from *P. minus* leaf tissue. After the leaf samples were collected from the INBIOSIS experimental plot, a six-hour hydro-distillation procedure was performed to extract the essential oil, which was then subjected to GC–MS analysis. Thus, this data could provide a reference and benchmark for researchers conducting analyses on volatile compounds in *P. minus*.

Specifications Table

**Table:** 

Subject	Biological Sciences
Specific subject area	Metabolomics and Natural Products
Data format	Raw .D data files and processed data in .xml
Type of data	Raw and processed GC–MS data.
Data collection	Mass spectrometry data was obtained from the Agilent 5975C inert MSD with a triple-axis detector mass spectrometer system (MS), coupled with the Agilent 7890A gas chromatograph (GC) equipped with a DB-5MS UI column (Agilent Technologies, USA). The data was processed and analysed using the XCMS online platform version 3.7.1.
Data source location	Universiti Kebangsaan Malaysia, Bangi, Selangor, Malaysia (2°55′09.0″N 101°47′04.8″E).
Data accessibility	Repository name: EMBL-EBI MetaboLights database Data identification number: MTBLS9155 Direct URL to data: www.ebi.ac.uk/metabolights/MTBLS9155 Instructions for accessing these data: The raw dataset is available online in the RAW FILES folder, while the processed data is accessible in the DERIVED FILES folder or Supplementary Table 1 provided in this article.

## 1 Value of the Data

• GC–MS data enables the identification of highly distributed volatile compounds which could act as the chemical marker for *P. minus.*
• Provide an overview of different classifications of volatile compounds that are potentially linked to various biological activities.
• Serve as a reference and benchmark for researchers conducting analyses on volatile compounds in essential oils, particularly those related to *P. minus.*

## 2 Background

*Polygonum minus* Huds, commonly referred to as kesum, is a traditional medicinal plant extensively utilized in Malaysia [1]. Beyond its palatable qualities, *P. minus* also possesses its distinct aroma, comprising easily volatile compounds. *P. minus* essential oil boasts a diverse range of advantages, including antioxidant, antimicrobial, and anticancer properties, as documented by [[2], [3], [4]]. Therefore, we performed GC-MS analysis on the essential oil extracted from *P. minus* leaf tissue to identify volatile metabolites associated with various biological activities. Then, MS data were analysed and processed using XCMS online version 3.7.1. Hence, this data could serve as a reference and benchmark for other researchers conducting studies on *P. minus* volatile compounds. The raw data (.D) and the processed data (.xml) were submitted to the EMBL-EBI MetaboLights database (www.ebi.ac.uk/metabolights/MTBLS9155), an open-access repository dedicated to metabolomics studies [5].

## 3 Data Description

This dataset consists of five replicates of mass spectrometry raw data (.D) files representing the essential oil extracted from *P. minus* leaf tissue. Subsequently, this dataset was analysed and processed using the XCMS online platform, version 3.7.1. The parameters were adjusted according to GC–MS parameter #350, with feature detection using the matchedFilter method: the signal-to-noise (S/N) threshold was set to 10, the maximal tolerated *m/z* deviation in consecutive scans was set to 30 ppm, the minimum peak width was set to 10, and the maximum peak width was set to 30. Then, both the raw data (.D) and the processed data in .xml format (Supplementary Table 1) were deposited to the EMBL-EBI MetaboLights database (www.ebi.ac.uk/metabolights/MTBLS9155). The raw data is available in the RAW FILES folder, while the processed data is available in the DERIVED FILES folder. The key finding of this analysis is the presence of aliphatic aldehydes—dodecanal (retention time: 18.28) and decanal (retention time: 13.54) —along with β-caryophyllene (retention time: 18.32), a sesquiterpene, as the highest abundant metabolites identified in *P. minus* essential oil (Supplementary Figure 1) [1,6].

## 4 Experimental Design, Materials and Methods

### 4.1 *Polygonum minus* cultivation and identification

The *P. minus* samples were propagated via stem cutting and grown in the INBIOSIS experimental plot (2°55′20.4″N 101°47′19.1″E) [7]. The samples were originally collected from Blue Valley, Cameron Highland, Malaysia (4°35′19.0″N 101°25′00.5″E). To further confirm its authenticity for this experiment, a *P. minus* plant sample was deposited at the UKM herbarium and verified by the UKM botanist with voucher number (ID007/2021).

### 4.2 *Polygonum minus* sampling and essential oil (EO) extraction

Fresh *P. minus* leaves were collected between 9–11 a.m. and 300 g of the leaves were ground in a commercial grinder with 1 L of distilled water The suspension was poured in a round bottom flask and extracted via hydro-distillation using a Clevenger-type apparatus for 6 h [8]. The EO was collected, then pooled together (1.9.2021–1.10.2021), and kept at −80 °C until further analysis.

### 4.3 Gas chromatography-mass spectrometry (GC–MS) analysis of *P. minus* essential oil

The *P. minus* EO (2.5 µL) was diluted with 1 mL hexane before the GC-MS analysis. The sample was profiled according to [8] with slight modifications using Agilent 7890A gas chromatography (GC) directly coupled to the mass spectrometer system (MS) of an Agilent 5975C inert MSD with triple-axis detector equipped with a 30 m, 0.25 mm, film thickness of 0.25 μm capillary DB-5MS UI column (Agilent Technologies, USA). The column temperature was set initially at 50 °C for 2 min and then gradually increased 6 °C/min to 280 °C for 10 min with the injector temperature at 250 °C and injection volume at 1 μL (split-less), while the transfer temperature was 290 °C. This analysis was conducted with 5 technical replicates.

### 4.4 Mass spectrometry data processing

The GC–MS raw data underwent conversion to the .CDF file format using MSD Chemstation. Subsequently, the converted data were processed and analysed using the XCMS online platform version 3.7.1, which encompasses feature detection, retention time correction, alignment, and annotation [9,10]. The data was assigned to a single job analysis and the parameters were modified based on GC-MS parameter #350, using the matchedFilter method for feature detection. Specifically, the signal-to-noise (S/N) threshold was established at 10, the maximum tolerated *m/z* deviation in consecutive scans was set to 30 ppm, and both the minimum and maximum peak widths were set to 10 and 30, respectively. Then, the raw data (.D) and the processed data (Supplementary Table 1) were submitted to the EMBL-EBI MetaboLights database (www.ebi.ac.uk/metabolights/MTBLS9155), an open-access repository dedicated to metabolomics studies [5].

## Limitations

None.

## Ethics Statement

The authors have read and followed the ethical standards for publication in Data in Brief. Henceforth, we affirm that the present study does not involve human subjects, animal experiments, or any data collected from social media platforms.

## CRediT Author Statement

**Muhammad Aqil Fitri Rosli**: Writing - original draft, Data curation, Formal analysis, Investigation, Methodology, Visualisation. **Sharifah Nabihah Syed Jaafar**: Writing - review & editing, Conceptualization**. Kamalrul Azlan Azizan**: Writing - review & editing, Methodology, Resources, Software, Validation. **Salmah Yaakop:** Writing - review & editing, Conceptualization**. Wan Mohd Aizat:** Supervision, Funding acquisation, Project administration, Resources, Writing - review & editing.

## Data Availability

MetaboLightsMass spectrometry data on volatile compounds of Polygonum minus Huds. leaf essential oil (Original data).

## Appendix Supplementary materials

Image, application 1

Image, image 2

## Footnotes

Supplementary material associated with this article can be found, in the online version, at doi:10.1016/j.dib.2025.111871.
