# Supplementary material for: Mass spectrometry data on volatile compounds of Polygonum minus Huds. leaf essential oil
Source: Data Brief. 2025 Jul 10;61:111871. doi: 10.1016/j.dib.2025.111871 (PMC12329244; doi:10.1016/j.dib.2025.111871)
Supplement: Supplementary file 1 [file mmc1.pdf]

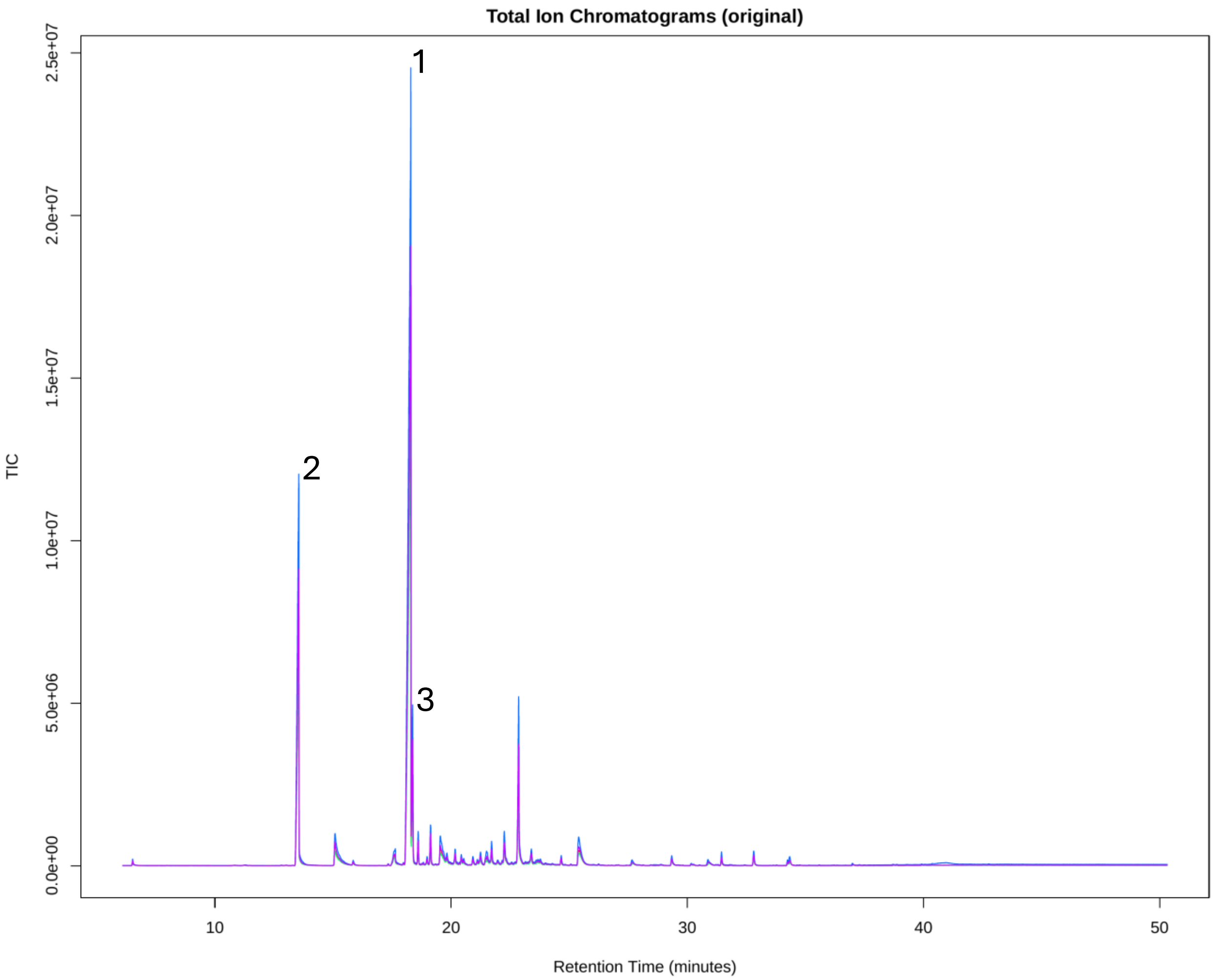

Supplementary Figure 1: Overlaid total ion chromatograms of five *P. minus* essential oil replicates generated using XCMS. The three most abundant metabolites were identified through the NIST library as: (1) dodecanal (retention time: 18.28), (2) decanal (retention time: 13.54), and (3) β-caryophyllene (retention time: 18.32).
